# Construction and validation of the APOCHIP, a spotted oligo-microarray for the study of beta-cell apoptosis

**DOI:** 10.1186/1471-2105-6-311

**Published:** 2005-12-29

**Authors:** Nils E Magnusson, Alessandra K Cardozo, Mogens Kruhøffer, Decio L Eizirik, Torben F Ørntoft, Jens L Jensen

**Affiliations:** 1 Molecular Diagnostic Laboratory, Department of Clinical Biochemistry, Aarhus University Hospital, Denmark; 2 Department of Theoretical Statistics, Department of Mathematical Sciences, Aarhus University, Denmark; 3 Laboratory of Experimental Medicine, Université Libre de Bruxelles, B-1070 Brussels, Belgium

## Abstract

**Background:**

Type 1 diabetes mellitus (T1DM) is a autoimmune disease caused by a long-term negative balance between immune-mediated beta-cell damage and beta-cell repair/regeneration. Following immune-mediated damage the beta-cell fate depends on several genes up- or down-regulated in parallel and/or sequentially. Based on the information obtained by the analysis of several microarray experiments of beta-cells exposed to pro-apoptotic conditions (e.g. double stranded RNA (dsRNA) and cytokines), we have developed a spotted rat oligonucleotide microarray, the APOCHIP, containing 60-mer probes for 574 genes selected for the study of beta-cell apoptosis.

**Results:**

The APOCHIP was validated by a combination of approaches. First we performed an internal validation of the spotted probes based on a weighted linear regression model using dilution series experiments. Second we profiled expression measurements in ten dissimilar rat RNA samples for 515 genes that were represented on both the spotted oligonucleotide collection and on the *in situ*-synthesized 25-mer arrays (Affymetrix GeneChips). Internal validation showed that most of the spotted probes displayed a pattern of reaction close to that predicted by the model. By using simple rules for comparison of data between platforms we found strong correlations (r_median_= 0.84) between relative gene expression measurements made with spotted probes and *in situ*-synthesized 25-mer probe sets.

**Conclusion:**

In conclusion our data suggest that there is a high reproducibility of the APOCHIP in terms of technical replication and that relative gene expression measurements obtained with the APOCHIP compare well to the Affymetrix GeneChip. The APOCHIP is available to the scientific community and is a useful tool to study the molecular mechanisms regulating beta-cell apoptosis.

## Background

Type 1 diabetes mellitus (T1DM) is an autoimmune disease caused by the selective destruction of the pancreatic beta-cells causing impaired insulin secretion. Beta-cell dysfunction and death in T1DM is the result of direct contact with activated macrophages and T-lymphocytes, and/or exposure to soluble mediators secreted by these cells, such as cytokines, oxygen free radicals and nitric oxide (NO) [1]. There is increasing evidence that apoptosis is the main cause of beta-cell death at the onset of T1DM [1-4] and after islet transplantation [1,5,6] Apoptosis is a regulated process, affected by expression of diverse pro- and anti-apoptotic genes [1,7,8] Cytokines play a role in the inflammatory destruction of islet grafts immediately after transplantation [9-11] a process that hampers the success of islet transplantation in patients with T1DM. In vitro beta-cell exposure to the cytokine interleukin (IL)-1β induces functional impairment, whereas exposure to IL-1β in combination with interferon (IFN)-γ and/or tumor necrosis factor (TNF)-α, induces beta-cell death by apoptosis in rodent and human islet cells after a period of 3–9 days [1-3] These cytokines modify the expression of several hundreds of genes in beta-cells, including stress response genes that are either protective or deleterious for beta-cell survival, whereas genes related to differentiated beta-cell functions are mostly down-regulated [12,13]

DNA microarrays have become a standard tool for several applications in molecular biology and provide a way to monitor the expression of thousands of genes in a single assay. The two major microarray platforms presently in use are the high density microarrays produced by *in situ *synthesis and the arrays produced by deposition of pre-synthesized DNA onto a solid surface. One widely used implementation is the Affymetrix GeneChip which uses photolithography and solid-phase chemistry to produce high density arrays of 25-mer oligonucleotides [14]. Spotted long oligonucleotides arrays were recently introduced as an alternative to cDNA arrays and in situ synthesized oligonucleotide arrays [15]. Utilizing this technology we have prepared a custom oligonucleotide array representing 574 genes chosen for their putative involvement in beta cell death, the APOCHIP. Gene selection was based on the analysis of a large number of array determinations of cytokine- and double stranded RNA-treated primary beta cells or insulin-producing INS-1 cells using Affymetrix chips [5,16-18]. This targeted and low cost array to be made freely available to the research community will allow the performance of detailed time-course studies and thus contribute to the understanding of the molecular events leading to beta cell dysfunction and death in diabetes mellitus.

To evaluate the performance of the spotted oligonucleotide array, we presently used two approaches. First we investigated the ability of the individual probes to respond to changes in target concentration. We expected that the M-value (log_2 _fold-change of test versus reference) would be proportional to the target concentration on a logarithmic scale and that slopes ideally would be close to one. We performed a weighted regression of M on concentration (log_2 _scale) using data from hybridisations at five different target concentrations. Next we used ten dissimilar RNA samples to compare the gene expression between the spotted array and Affymetrix platforms. We expected that this would yield a sufficient number of differentially expressed genes to allow for meaningful conclusions to be drawn about the concordance between the two platforms.

Our data suggest a good reproducibility for technical replications both within and between chips. High concordance to the Affymetrix GeneChip in terms of relative gene expression indicates that the APOCHIP is a reliable tool for studying the molecular mechanisms involved in beta cell apoptosis.

## Results

### The Model based approach

#### Internal replication

The results of the internal replicates are shown in Table 1. The spot variation was roughly 1.5 fold as large as the measurement variation. This shows that there are moderate variations in the replicated spots within a chip. The channel variation was of the same order of magnitude as the spot variation except for the lowest target concentration, where it was much larger (~3 fold). The origin of the channel variation remains to be clarified, but it may be due to intensity dependent properties between the two channels. By normalising the two channels against one another, block wise, we obtained only a small reduction in the channel variance (data not shown). The two chips with the lowest concentration generally showed higher variance values than at the higher concentrations, and may reflect that the target concentration for the test sample (1/3 μg/20 μL) is close to the lower detection limit of this system, a hypothesis supported by the substantial increase in "bad" and "not found" calls around this concentration (data not shown).

**Table 1:** Standard deviations for the various random terms in the log_2 _fold-change for all the chips in the dilution series.

concentration μg/20 μL	σω_m_	σ_s_	σ_c_	median f.c.	Observed concentration
1/3	0.10	0.12	0.36	0.53	0.35
	0.12	0.12	0.38	0.52	
1	0.09	0.13	0.18	1.54	1.00
	0.08	0.11	0.18	1.45	
2	0.08	0.14	0.17	2.53	1.72
	0.08	0.14	0.18	2.61	
3	0.08	0.12	0.17	4.03	2.72
	0.08	0.12	0.18	4.10	
4	0.08	0.13	0.19	4.60	3.00
	0.08	0.12	0.19	4.32	

The first column σω_m _is the average measurement error, the second σ_s _is the spot variation, and the third σ_c _is the channel variation. The fourth column is the median fold-change, exp [(ζ_1j _- ζ_2j_) ln(2)], between the two channels. The last column is the ratio of median fold-change to median fold-change for concentration 1.

#### External replication

Table 2 shows the estimated additional variance as compared to that predicted by the model when calculating log_2 _fold-changes from technical replications. Most of the estimates are negative, showing that there is no additional variance and indicating a good reproducibility for technical replication (Figure 2).

**Table 2:** Additional variance when determining fold-changes on two chips (technical replication).

concentration μg/20 μL	Extra variance	2σ^2^ω_m_^2^
1/3	-0.001	0.024
1	-0.001	0.015
2	0.003	0.012
3	-0.004	0.012
4	-0.003	0.012

The first column is the additional variance where a positive value indicates additional variance. The last column is the variance of the measurement error obtained from Table 1 and is included for comparison.

**Figure 1:**
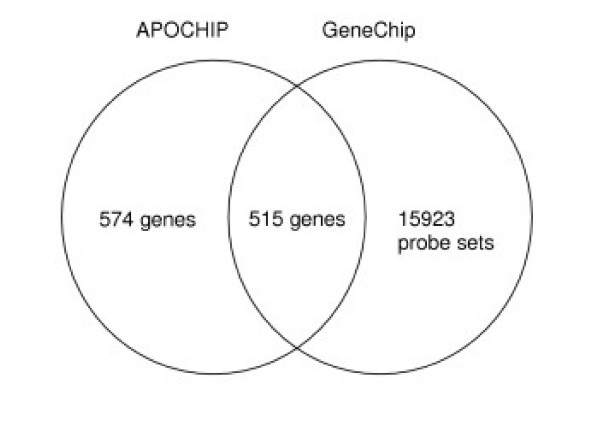
**Representation of the APOCHIP and GeneChip microarrays**. Representation of genes on the two array types illustrating the number of genes represented by at least one probe or probe set on each array type and the total number of genes present on the arrays. A total of 515 genes were represented on both array types.

**Figure 2:**
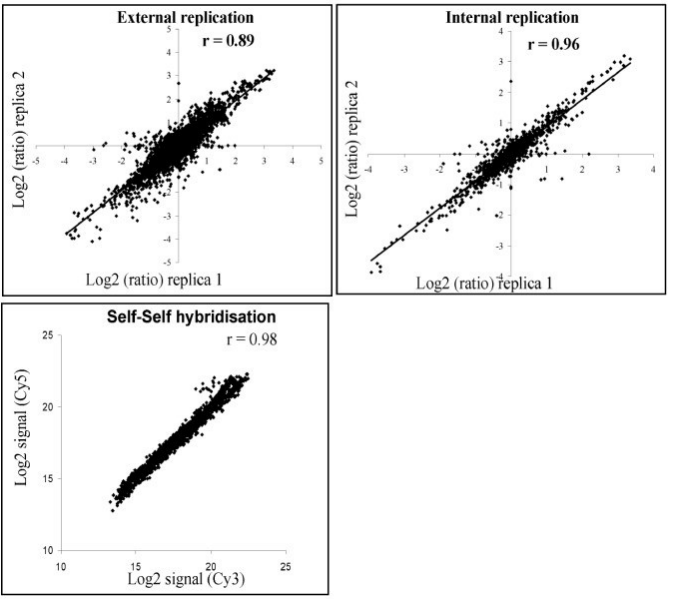
**Comparison of gene expression measurements illustrating the technical reproducibility of the spotted long oligonucleotide array**. The upper left subplot shows technical replication between spotted arrays within print batches. The upper right subplot shows technical replication between replicated spots within arrays. The lower left subplot shows a self-self hybridisation (the sample is labelled to both Cy3 and Cy5 separately and hybridised together) illustrating the reproducibility between Cy3 and Cy5 fluorofores. Data was normalised using a lowess transformation implemented in the MIDAS software. Pearson correlation coefficients (r) are shown for each comparison and are representative (mean) of at least four independent experiments. Lines are lines of equality.

The spot variance provides information on the difference between using a one-colour system as opposed to a two-colour system. In a two-colour system the spot variance terms for each channel within a chip cancel when using ratios. Thus a log_2 _fold-change obtained from two chips in a one colour system will have a variance of at least 2σ_s_^2 ^+ 2σ^2^ω^2 ^compared to a two colour system where it is 2σ_s_^2 ^(Table 3 and Additional file 2). As depicted in Table 3 the estimated one-colour variance was comparable to the spot variance when print-tip differences were accounted for in the normalisation.

**Table 3:** Variance in a one-colour system.

concentration μg/20 μL	One-color variance	One-color variance, block	2σ_s_^2^
1/3	0.093	0.056	0.027
1	0.057	0.053	0.029
2	0.101	0.044	0.040
3	0.048	0.030	0.027
4	0.046	0.033	0.031

In the first column a global median centering is used, and in the second a median centering for each of the 8 blocks is used. The last column gives 2σ_s_^2 ^for comparison.

#### Fold-change regression

**Figure 3:**
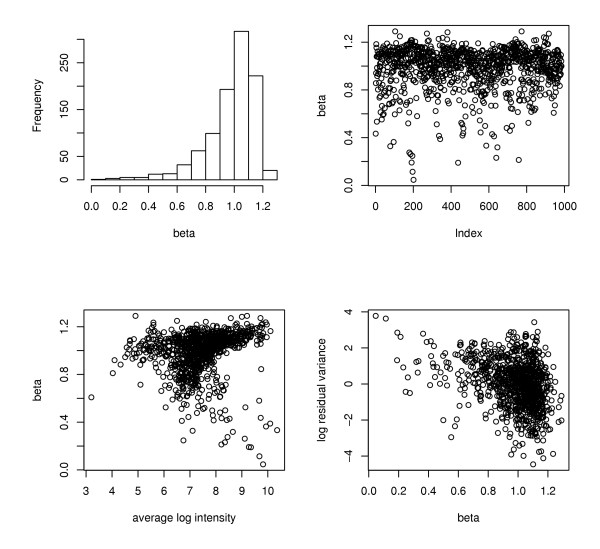
**Representation of the APOCHIP linear regression for the dilution series**. The upper left subplot shows a histogram of all the slopes from the linear regression, and the upper right subplot shows the slopes as a function of the position on the chip. The lower left subplot gives the slopes as a function of the average intensity. For high intensity values the slopes are either large or small. In the lower right subplot are the variance terms σ_f_^2 ^as a function of the slopes.

The result of the regression is illustrated in Figure 3. If the fold-change is proportional to the concentration the slope β in the regression of log_2 _fold-changes is 1. As can be seen in Figure 3 most slopes are in the range 0.8–1.2 (78%). Interestingly, there seems to be a spread around 1 suggesting that each gene has its own sensitivity to changes in the concentration. A formal test at level 5% for the slope being equal to one gives acceptance in only 25% of the genes. Furthermore, as the lower left subplot of Figure 3 shows, when the signal intensity is very high all slopes are either larger than 1 or very small. Also, a small value of the slope does not imply that the probe does not respond at all, rather the sensitivity to changes in the concentration is limited. The physical origin of these phenomena is unclear. Using the linear relation between the measured log_2 _fold-changes and the log_2 _concentration we may ask for which probes are we able to detect a true fold-change of a certain size. Using average properties and considering only internal replication we found in our experiments that for a true log_2_fold-change λ the measured log_2 _fold-change is roughly normally distributed with mean βλ and standard deviation 0.09. If we want the measured log_2 _fold-change to be larger than αλ, for some chosen value of α, the probability of this event is the probability that a standard normal variate is bigger than (α-β) λ/0.09. As an example, if we require this latter probability to be bigger than 0.5, we find that we can use the probes with β > α. Elaborating on this example, if we take α = 0.5, there are in our experiment 97 % of the probes satisfying β > 0.5, with 34 probes only left out.

As depicted in Table 1 we observed a discrepancy between the log_2_ concentration and the median log_2_ fold-change. This may partly be accounted for by the scanner settings which were set to fixed but arbitrary values. Considering the self-self hybridisations (concentration 1, Table 1) it is evident that the settings for the test channel were too high compared to the settings for the reference channel. This effect may be minimized by using automated settings generated by the scanning software (data not shown). However, the ratios between two consecutive concentrations are close to the expected values except for the highest concentration where it is lower than expected (Table 1).

### Cross platform comparison

We compared the relative expression of 515 genes present on both the APOCHIP and Affymetrix GeneChip 230A arrays. These genes, corresponding to 949 probes on the APOCHIP, were used to compare the relative gene expression profiles in ten rat RNA samples. On average, 93 % of the spots were called "good" by the Scanarray Express software and 7 % was called either "bad" or "not found". The samples and the pooled reference was analysed separately on GeneChip 230A arrays, since this system utilizes single colour hybridisations. Normalised M-values (sample vs. reference) were calculated for each probe set on the array using RMA [19] and Affymetrix MAS 5.0 algorithm that compares signal intensity from perfect-match and mis-match 25-mers [14]. On average, 65 % of the genes surveyed on these arrays were called "present" and 34 % were called "absent" and the remainder "marginal" using MAS 5.0. This software also reports calls for "increased" (I), "decreased" (D) and "no change" (NC) for the relative gene expression. To take into account possible differences due to normalisation methods we compared the results obtained by our approach (MAS 5.0/median centering) to those obtained using RMA and a LOWESS (LOcally WEighted Scatterplot Smoothing) procedure implemented in MIDAS [20]. We found similar results particularly when low intensity data was excluded, as described below (data not shown).

**Figure 4:**
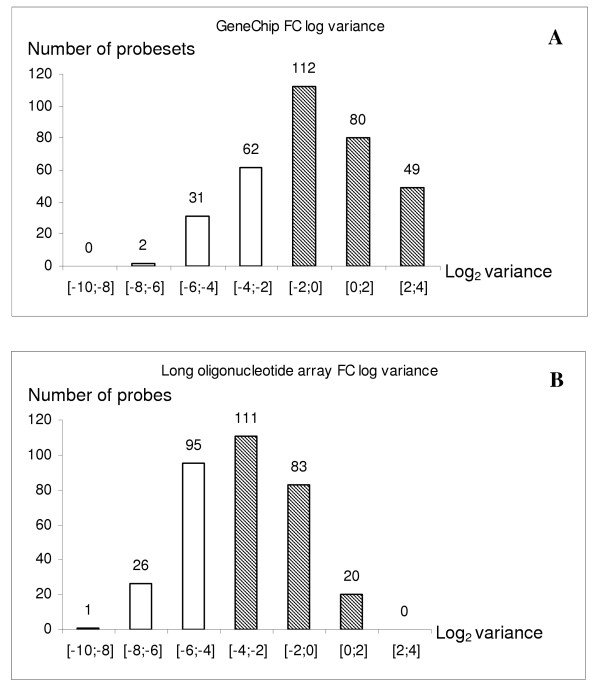
**Distribution of probes according to log_2 _fold-change variation**. Representation of the distribution of probes according to log_2_fold-change variation (on a log_2 _scale) within the 10 arrayed RNAs. A. GeneChip B.APOCHIP. Genes exhibiting a variation > -2 (Affymetrix) and > -4 (APOCHIP) were included in the comparison.

**Figure 5:**
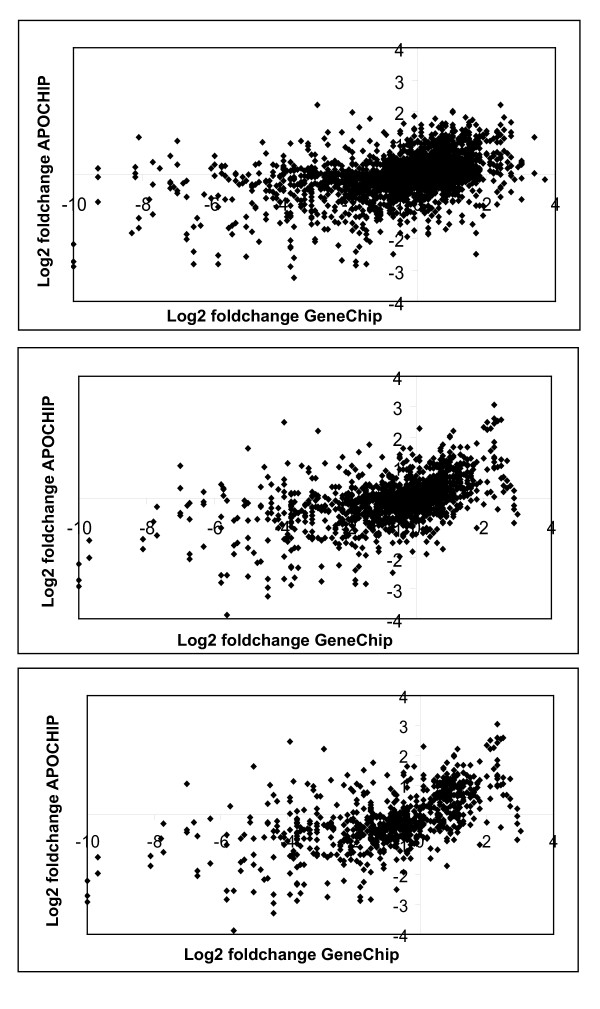
**Cross-platform comparison illustrating the correlations between the APOCHIP and the Affymetrix array**. The upper left subplot illustrates the raw data. The upper right subplot corresponds to data were low intensity data was removed. The lower left subplot corresponds to the most varying genes in the comparison.

As low intensity data are prone to increased variation [21] and therefore less reliable we set the following criteria for the comparison: a. Affymetrix array: 1. For "NC" calls both test and reference pool signal should be called "present", 2. For "I" calls the test signal value should be called "present", 3. For "D" calls the reference pool signal should be called "present"; b. APOCHIP: Measurements associated with "not found" or "bad" were excluded. We then focused on the remaining 496 probes that fulfilled the above criteria in all ten measurements on both platforms. The results are listed in Table 4 and illustrated in Figure 4 and Figure 5.

**Table 4:** Representation of the cross-platform comparisons.

Criteria	Probes/Genes	Coefficient of correlation r_median_
1. All data	949/515	0.39
2. No data associated with absent, bad, not found	496/308	0.64
3. Most varying probes	267/164	0.84

The first column show the different steps of the data filtering, the second show the number of probes and genes compared, and the third show the median coefficient of correlation.

Without this quality filtering of the probes the median of the weighted Pearson correlation was 0.39, whereas the filtering increased this value to 0.64 (first two lines of Table 4). A further filtering of the probes may be relevant. If a gene has no differential expression between the ten samples there is no possibility of estimating the correlation. Similarly, if the probe does not respond at all in one of the two platforms, the estimated correlation is unreliable. In an attempt to avoid this we removed probes that had a low variation over the ten samples in either one or both of the two platforms. The Affymetrix GeneChips showed the largest range of the log_2 _ratios. To compare a large number of probes and include only the most varying we set an arbitrary cut-off of 0.25 for the Affymetrix platform. To include a similar number of probes for the APOCHIP we set an arbitrary cut-off of 0.0625 for the variance of this platform. This reduced the number of probes to 267 (164 genes) (Figure 4). For this reduced set of probes the median correlation was 0.84 (Table 4), indicating a tight concordance between the two array types.

**Figure 6:**
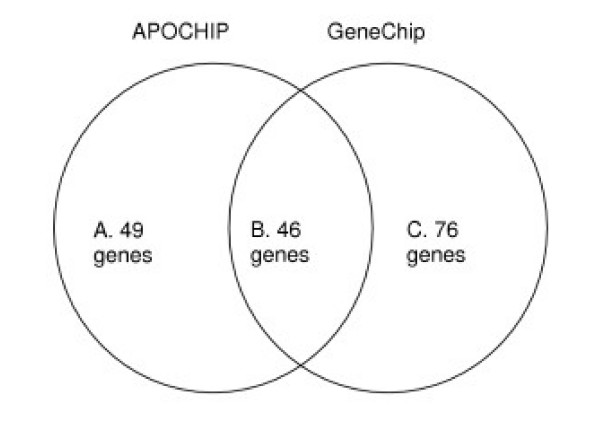
**Representation of the excluded genes that did not exhibit high variation of log_2 _fold-change (on a log_2 _scale) on both platforms**. A; Genes that showed high variation of fold-change only on the long oligonucleotide array (log_2 _fold-change variation: long oligonucleotide array > -4/GeneChip < -2). B; Genes exhibiting low variation on both platforms (log_2 _fold-change variation: long oligo array < -4/GeneChip < -2) C; Genes exhibiting high variation of fold-change only on the GeneChip array (log_2 _fold-change variation: long oligonucleotide array < -4/GeneChip > -2).

The distribution of the genes excluded from the analyses is illustrated in Figure 6. Of the 164 most varying genes, 9 gave discordant results exhibiting a negative correlation (Table 5). Further analysis of these genes revealed that in most instances the signal intensities were below the mean signal intensity on either one or both platforms. Moreover, two of these genes displayed variations close to the lower limits for one or both platforms as described above, indicating that the correlations obtained for these genes may be less reliable (Table 5). To further address this issue we performed a BLAST [22] search based on the long oligonucleotide sequences. We then mapped these probes and corresponding Affymetrix probe-sets to the mRNA sequence on which the APOCHIP probe was based. Second, we checked for sequence overlap between the probes of the corresponding platforms. As depicted in Table 5 we found that six of the Affymetrix probe-sets mapped to the APOCHIP mRNA but only one probe-set (Affymetrix ID: 1367713_at) showed overlap with the APOCHIP oligonucleotide probe sequence. For two of the remaining probes the Affymetrix probes did not properly match the APOCHIP mRNA, suggesting that these sequences interrogate different sequences for these genes. For the last APOCHIP probe (GenBank ID: XM_213699) the mRNA annotation was changed and the 60-mer did not match the transcript perfectly. In cases where both platform sequences align perfectly to the APOCHIP mRNA other factors such as differences in specificity and sensitivity, RNA splice variants, and RNA structure of the probes may be important.

**Table 5:** Representation of the spotted probes exhibiting negative correlation coefficients with Affymetrix probes.

Long oligonucleotide sequence 5'-3'	GenBank accession no.	Probe Set ID RAE230A	Correlation coeficient
CACAGAAGATGGAGAAAATCTAAAG-TGAAAGTGCGCGTGACACACATGCA	^c^XM_213699 *NM_001011901	^c^1388898_at	-0.37107
CAGACCTTCATCGCTCTGTGTGCTACCGA-CTTCAAGTTTGCCATGTACCCGCCATCGA	^c^D16308	^c^1370810_at	-0.24892
GGACATCTGAGTTGAGACCCAGTTGTTAC-TAACCTTATTGTGAATTGCCTGATCTACA	^a,b^M11794	^a,b^1371237_A_AT	-0.13854
ATTCTCTGGTCTAATGTCTGGCTGGGGT-TCTCCGTCTGCTTCCTGTATCTATATTCT	^b^M15562	^b^1370883_AT	-0.43223
ACTTACAAGGACCAAATACCAAACTAGAAG-AAAAGATAGACATGGTGCCTATTGACTTTC	^c^M63101	^c^1387835_AT	-0.93746
CTAAAATTGGGCTTGCGGTTTTCATTTCTG-ATGTCTCTGGATTGGCACCCTTATGGTTTA	^a,b,d^NM_019356	^b,d^1367713_at	-0.88595
GTCTGTAAAATAACCATCAGTTCCTTCAC-CCACCCTCTTCCCCTTAACACTCAGC	^b^XM_340904 *NM_012809	^b^1387897_at	-0.6239
TGTAAATTACACCACGGACTTCATCTACC-AGCTCTACTCAGAGGAAGGGAAAGGAG	^a,b^L25387	^b^1372182_at	-0.37801
GTCTTGACAATGTTCACAAACAGAGAGT-GGCTGAAGTGCTAAATGACCCTGAGAA	^a,b,d^XM_216792 *XM_343114	^a,b^1370886_a_at	-0.82769

a. Low signal intensity (signal below mean of all arrays); b. Affymetrix and long oligonucleotide probes mapped to the APOCHIP target mRNA; c. Affymetrix and long oligonucleotide probes mapped partly or not at all to the APOCHIP target mRNA; d. Probes exhibiting variation close to the lower limits shown in Figure 4. *. New annotation obtained by BLAST.

## Discussion

Microarrays have been widely used for expression profiling [14,23], discovery of gene function [24,25], pathway dissection [26], classification of clinical samples [27,28] as well as investigation of RNA splice variants [29]. Several studies have been conducted comparing gene expression across platforms with varying results [30-39]. Whereas quantitative RT-PCR are usually found to agree well with corresponding array data concerns have been raised in some studies comparing different array formats [29,32,33,37]. Thus, Kuo et al. [32] compared cDNA and Affymetrix 25-mer arrays and reported little concordance. The data in this study, however, was originated from two different laboratories and it is not clear whether the poor agreement was due to differences in the array types. Moreover, these results were based on absolute measurements which may be misleading [40]. Li et al. [29] and Kothapalli et al. [33] also used cDNA and Affymetrix arrays and in both cases found substantial discrepancies; based on these findings, it was inferred that cDNA arrays often fail to identify differentially expressed genes. On the other hand, strong support for the use of long oligonucleotide microarrays comes from two independent studies [30,34], and several recent studies suggest a robust concordance between the different microarray platforms [40-42]. Hughes et al. [30] reported high concordance utilizing data from 60-mer oligonucleotide arrays synthesized by an ink-jet oligonucleotide synthesizer, cDNA arrays and Affymetrix GeneChip arrays. Barczak et al. [34] compared relative gene expression measurements of a large collection of spotted 70-mers against Affymetrix GeneChips and found good agreement.

Although, the majority of the most differentially expressed probes yielded high correlations, there were exceptions (Table 5). There was also a group of genes exhibiting relatively large log_2_ fold-change variation in one, but not the other, platform (Figure 6). These findings may partly be explained by differences in sensitivity and specificity and other probe specific effects. Of note, in some cases differences in transcript annotation and/or RNA splicing may be more important than discrepancies in array performance. Several factors may influence the reproducibility when comparing data across platforms. Proper gene identification is essential as genes can only be compared if they are accurately identified on both platforms [43]. This can be difficult as transcript information often comes from different sources and are continuously being improved. The starting material must be consistent and procedures for RNA handling standardized. There are several labelling procedures in use, amplification versus no amplification, direct versus indirect dye incorporation which may contribute to downstream biases [43]. In this study the samples were treated identically prior to RNA amplification and similar amplification and labelling protocols were used for both array types. Pre-processing and methods for data handling may also influence the final results [44]. As stated in the Results section, there were differences using different spot identification software and normalisation algorithms, but these differences were substantially reduced by removing low intensity data and by comparing only the most varying genes (data not shown). Moreover, when comparing gene expression data across platforms it is essential to do so using relative measurements, since absolute measurements are affected by probe and platform specific properties that may cause misleading interpretations [40]. As discussed above, low signal intensities are prone to increased variation [21] a phenomenon that is well established for most array formats, including spotted 30 mer arrays [45], in situ synthesized 24 mer arrays [46] and GeneChips [47,48]. Thus, it was not surprising to find that the correlation between differential measurements improved significantly when low-intensity measurements were excluded. Although intensities between two identical samples labelled to different dyes are rarely equal across all spots, we find that much of this variation is removed after proper normalisation (Figure 4 subplot 3). Two-colour hybridisations are generally used for spotted arrays, and many study designs involve comparison of the test sample to a common reference sample. Accurate quantification of a particular gene requires that the reference sample contains sufficient RNA to produce a clear signal for the corresponding probe. Reference samples may be generated from a pool of several cell lines, or as here, by pooling of all samples obtained from different tissues. The rationale for pooling the samples is that differentially expressed transcripts will also be present in the reference. Reference pools may not always produce sufficient signal intensity to allow for accurate quantification of some of the probes. When using Affymetrix MAS 5.0 software to analyse the pool reference for the subset of genes associated to both platforms, 76 % of the probe sets were called "present", as compared to 65 % "present" calls on average in the present data. Different designs such as a reference-free setup where pairs of test samples are compared directly may be preferable depending on the application [49].

Oligonucleotide probe design may also be important for signal intensity and for measuring differential gene expression. Oligonucleotide probes are designed on the basis of sequence. Several criteria, such as GC content and melting point, are used in the design but it is not possible to accurately account for differences in structure which may lead to unwanted steric effects. We observed that there were sometimes large numerical differences in the signal intensity of different spotted probes corresponding to the same gene (data not shown) a phenomenon that has been noted by others [14,34]. In a few cases long oligonucleotides representing the same gene gave discordant results. Such differences between probes may depend on several factors, including low sensitivity of some probes, alternative splicing, nucleic acid structure, distance from the 3' end of the RNA transcripts, GC content, and cross-hybridisation to unknown or poorly characterized mRNAs including pseudo genes and non-coding RNAs. Hence, the use of standardised sets of probes and protocols is an important issue when data from different laboratories and array platforms are compared [40-42,50,51]. Selection of a suitable microarray platform is influenced by several considerations. The Affymetrix system has been widely used for several applications and holds the advantage of standardisation in terms of probes and hybridisation protocols and, to some extent, data quantification [40]. However, this technology has been limited by cost considerations for projects involving a large number of samples. Spotted arrays are labour intensive, but they can be made in large quantities by individual laboratories at a lower cost. Moreover, sequences with high homology to other genes can be avoided and probes for novel genes and gene variants may readily be designed.

In conclusion, we have constructed and validated the APOCHIP, a spotted microarray designed for the study of beta cell death in diabetes mellitus that may be of use to the scientific community. Designing and printing in-house arrays offers a flexible mean to carry out combinations of extensive multipoint and detailed time course gene expression analysis, following exposure of pancreatic beta-cells to different pro-apoptotic stimuli. We expect that this array will help research in the field enabling the performance of more detailed and complete experiments.

## Conclusion

We have validated a rat oligonucleotide microarray constructed for the study of beta cell death in diabetes mellitus. We evaluated the technical reproducibility of the array by estimating the variance associated with the internal and external replication. We then used a fold-change regression model to estimate the ability of the probes to respond to changes in target concentration. Finally, we used ten dissimilar RNA samples to compare the relative gene expression between the spotted array and Affymetrix platforms. We found a high reproducibility for technical replications both within arrays and between arrays, with most oligonucleotide probes responding to target concentration in a manner close to that predicted by the model. There was a clear relation between successive data filtering and concordance between the two array types; by comparing only the most variable genes on both platforms we found that there was a high concordance between the APOCHIP and the GeneChip platform, supporting the validity of this approach.

## Methods

### Isolation of total RNA

Total RNA was isolated from snap frozen cells and tissue using Trizol. Each sample was dissolved in 1 mL Trizol^® ^reagent (Invitrogen) on ice and homogenised using a Fastprep homogeniser (Bio 101 Savant Instruments Inc.) according to the manufacturer's instructions. Trizol was removed by addition of chloroform followed by isopropanol precipitation. The precipitates were washed using 75 % ethanol. The amount and purity of RNA was quantified photo-spectrometrically by measuring the optical density at 260 and 280 nm and the integrity was checked by agarose gel electrophoresis.

### cRNA preparation

#### Affymetrix arrays

For each hybridisation reverse transcription was performed on 5 μg total RNA for 1 hour at 42°C using a T7 oligo(dT)_24_-primer and reverse transcriptase (SuperScript II; Life Technologies Inc.). Second-strand cDNA synthesis was performed for 2 hours at 16°C using *Escherichia coli *DNA polymerase I, DNA ligase, and RNase H (Life Technologies Inc.) followed by incubation in 50 mM NaOH and 0.1 mM EDTA for 10 minutes at 65°C to degrade the RNA. After phenol-chloroform extraction and ethanol precipitation, in vitro transcription was performed for 6 hours at 37°C using biotin-16-UTP and biotin-11-CTP with an RNA transcript labelling kit (BioArray; Enzo Diagnostics). cRNA was purified on RNeasy spin columns (Qiagen), followed by fragmentation for 30 minutes at 95°C.

#### Spotted oligonucleotide chip

Total RNA extraction, reverse transcription on 5 μg total RNA and second strand cDNA synthesis were performed as described above. In vitro transcription was performed for 6 h at 37°C using amino-allyl-UTP and T7 Megascript Kit (Ambion). The produced cRNA was purified using Rneasy spin columns (Quiagen) followed by coupling of Cy3 and Cy5 fluorescent dyes in water-free DMSO for 2.5 h at room temperature. The labelled cRNA was fragmented for 30 min at 60°C in a 50 mM ZnCl_2 _solution and excess dyes were removed by ethanol precipitation of the cRNA.

### Spotted oligonucleotide microarray procedures

#### Oligonucleotide probe design

The genes on the spotted array were selected based on our large data set obtained with GeneChip (Affymetrix) analyses of two different treatments that induce beta cell apoptosis, namely cytokines and double stranded RNA [13,16-18]. We used three criteria to select genes to grid in our custom microarray: First, largest numerical alterations in gene expression; Second, representing informative gene clusters (e.g. genes involved in NO production, signal transduction/transcription factors, bcl-2 family, ER stress, etc); Third, genes showing distinct expression patterns over a time course (identified by self organizing maps). The complete list of genes present in the APOCHIP is provided in Additional file 1. Moreover a number of genes were selected for normalisation purposes. These genes were chosen to cover a range of signal intensities from low, medium to high. For each gene on the array one to three 60-mer oligonucleotides were designed using the Array Designer software (Premier Biosoft International).

#### Hybridisation, washing and scanning

The probes were spotted in duplicate on Codelink slides (Amersham Biosciences Inc.) at 30 % relative humidity and 20°C using a VersArray Chipwriter from BioRad. For a standard hybridisation one μg of each Cy3 and Cy5 labelled target sample was applied to the microarray slide in a volume of 20 μL for 16 h at 42°C. Before scanning all slides were washed as previously described [30]. The two replicates were spotted below one another on the chips and all hybridisations were carried out twice on separate arrays. The samples were labelled with Cy3 and a common reference pool was labelled with Cy5. Following scanning of the glass slides the fluorescent intensities were quantified and background adjusted using an "adaptive circle" method implemented in the Scanarray Express software (PerkinElmer). Data was normalised by a blockwise median centering within individual hybridisation pairs and mean log_2_-expression ratios were calculated from the four measurements of each probe. Probes exhibiting expression values higher than 60000 (arbitrary units) in one chip within any comparison were discarded from the analyses. Probes exhibiting negative expression values in more than four chips were discarded from the analyses and remaining negative values were set to 1.

### Experimental design

#### A model-based approach for internal validation of spotted oligonucleotide probes

##### Dilution series hybridisations

Total RNA and cRNA from rat kidney, heart, liver, and muscle tissue was prepared as described above. Equal amounts of cRNA from all samples were pooled and divided for fluorescent labelling to the dyes Cy3 and Cy5 as described above. Hybridisations were performed at five concentrations of Cy3 labelled target (0.3 μg/20 μL, 1 μg/20 μL, 2 μg/20 μL, 3 μg/20 μL, 4 μg/20 μL). The Cy5 material was used as reference and was kept at constant concentration of 1 μg/20 μL in all hybridisations. Arrays were scanned at identical laser (100 %) and PMT (50 for Cy5 and 65 for Cy3) settings.

In the spotted array the total variation contains contributions from: a. variations in the spots; b. variations in the two channels; c. variations between arrays. To study the variation in the system we modelled the log_2 _expression value x_gcj _for gene g, channel c = 1, 2, and internal replicate j = 1, 2 as a sum of terms representing the different variations. Terms that are used to model the mean value structure are denoted levels and terms that are used to model the variance structure are called random. We wrote the log expression as a gene level (μ_g_), plus an overall channel and replication level (ψ_cj_), plus a random spot variation (u_gj _with variance σ^2^_s_), plus a random gene specific channel difference (υ_gc _with variance σ^2^_c_), plus, finally, a random measurement error (ε_gcj _with variance σ^2^ω_gcj_^2^, where ω_gcj_^2 ^is a known term). Here σ^2^_s _(s for spot) reflects the difference in morphology of the spots and is not related to the gene. Similarly, σ^2^_c _(c for channel) reflects that the two channels react differently depending on the gene, the variation in this gene specific channel difference is then given by σ^2^_c_.

Mathematically we write the model as x_gcj _= μ_g _+ ξ_cj _+ u_gj _+ λ_gc _+ ε_gcj_. As suggested by Churchill et al. [52], we model some of the variation as random components. To take into account the larger variances associated with small expression values [21] we scaled the variances using the standard deviations s_gcj _for the pixel intensities of each spot supplied by the software. Transforming s_gcj _to the log_2 _scale we used ω_gcj _= s_gcj_/[exp(x_gcj _ln(2))ln(2)]. The overall levels x_icj _were estimated by median values and we let y_gcj _= x_gcj_-x_icj _be the remainder when the estimated overall level was subtracted.

##### Internal replication

We first considered the variance of the measurement error. The measurement variance can be evaluated by looking at the difference d_g _= (y_g11 _- y_g21_) - (y_g12 _- y_g22_) between the two log_2 _fold-changes corresponding to the internal replication. The variance of this difference is σ^2^s_g_^2 ^where s_g_^2 ^is the sum of the four terms of ω_gcj_^2 ^for gene g. A natural estimate for σ^2 ^is then the average of the squared scaled differences d_g_/s_g_.

Having estimated the measurement variance we could next estimate the spot variance σ_s_^2 ^and the channel variance σ_c_^2^. For the spot variance we considered the sum over the two channels of the difference between the two replicates: (y_g11 _- y_g12_) + (y_g21 _- y_g22_). The variance of this term is 8σ_s_^2 ^+ σ^2^s_g_^2^, and having found the measurement variance σ^2 ^above we then used the observed variance of these terms to estimate the spot variance σ_s_^2^. Similarly, for the channel variance we considered the sum over the two internal replicates of the log_2 _fold-changes (y_g11 _- y_g21_) - (y_g12 _- y_g22_), which has variance 8σ_c_^2 ^+ σ^2^s_g_^2^. As above we estimated σ_c_^2 ^from the observed variance of these terms.

##### External replication

To examine the reproducibility of the external replication we calculated a log_2 _fold-change for each of the two chips and considered the difference of these. We compared the variance of these differences with that predicted by the model.

##### Fold-change regression

For each probe and concentration we calculated a common log_2 _fold-change from the two internal and the two external replicates. The variances of these are τ_g_^2 ^r_gi_^2^, where g is gene and i is concentration, and where r_gi_^2 ^is given through σ^2^ω^2 ^above. Next, for each gene we performed a regression of log_2 _fold-change against the median of the log_2 _fold-changes, where the factor τ_g_^2 ^in the variance describes how well the linear relation fits the data.

##### Cross platform comparison of gene expression

Total RNA and double stranded cDNA from ten dissimilar rat tissues were prepared as described above. To minimise the variation associated with preparation of double stranded cDNA, each sample of double stranded cDNA was divided in two equal volumes that were used to prepare cRNA for hybridisation to Affymetrix GeneChips-RAE230A and for hybridisation to the spotted arrays.

##### Affymetrix arrays

A common reference pool was prepared by pooling equal amounts of cRNA from all samples investigated. We analysed 10 samples and common reference cRNA on GeneChips RAE-230A (Affymetrix Inc.). These arrays were hybridised with 15 μg of labelled cRNA for 16 h at 45°C while rotating. The chips were stained in an Affymetrix Fluidics station with streptavidin/phycoerythrin, followed by staining with an antistreptavidin antibody and streptavidin/phycoerythrin. The chips were scanned using a HP-laser scanner and the readings from the quantitative scanning were analyzed by the Affymetrix Gene Expression Analysis microarray Suite Software (MAS) 5.0. Each microarray was scaled to "150" as previously described [53]. Data was also normalised using the Robust Multiarray Analysis (RMA) normalisation approach in the Bioconductor Affymetrix package to the R project for statistical computing [19].

##### Spotted oligonucleotide chip

A common reference pool was prepared by pooling equal amounts of cRNA from all investigated samples. The reference pool was labelled to Cy5 and the ten samples were labelled to Cy3 as described above. For each sample one μg of each Cy3 and Cy5 labelled target was applied to the microarray slide. Data was normalised as described in the Hybridisation, washing and scanning section.

##### Comparison of relative gene expression between platforms

To identify genes common to both platforms we used a combination of publicly available databases, DAVID [54] and Affymetrix [55], to identify UniGene clusters (build 99) and GenBank accessions. Based on this information, we were able to compare gene expression measurements for 515 genes represented on both platforms (Figure 1). For each gene we calculated the correlation between the values from the two platforms using a weighted Pearson correlation. The weighted Pearson correlation is obtained from the usual Pearson correlation by replacing all the sums entering this formula by weighted sums, using the same weights as those used in the regression above (see Additional file 2). These weights were obtained from the spotted arrays and are also used for the GeneChip values.

## Authors' contributions

NEM initiated the present study and was responsible for the design and construction of the APOCHIP and for the handling of microarrays. TFØ and MK supervised the microarray procedures. NEM, AKC and DLE selected the genes for the APOPCHIP. JLJ did the mathematical/statistical work. NEM and JLJ interpreted the results and wrote the article. DLE, TFØ, and AKC made improvements and suggestions to the manuscript.

## Supplementary Material

Additional File 2 **Estimation of varation-detailed**. Detailed description of the variation in the APOCHIP two-colour system. Click here for file

Additional File 1 **Table S1**. Complete list of genes represented on the APOCHIP. Columns 1–12: Probe sequences and Gene annotations. Columns 13–22: Log_2 _fold-change GeneChip. Columns 23–32: Log_2 _fold-change APOCHIP. 33–41: Correlation coefficients, Log_2 _fold-change variation, and dilution series data. Click here for file
