# Supplementary material for: Construction and validation of the APOCHIP, a spotted oligo-microarray for the study of beta-cell apoptosis
Source: BMC Bioinformatics. 2005 Dec 29;6:311. doi: 10.1186/1471-2105-6-311 (PMC1368999; doi:10.1186/1471-2105-6-311)
Supplement: Additional File 1 — Table S1. Complete list of genes represented on the APOCHIP. Columns 1–12: Probe sequences and Gene annotations. Columns 13–22: Log2 fold-change GeneChip. Columns 23–32: Log2 fold-change APOCHIP. 33–41: Correlation coefficients, Log2 fold-change variation, and dilution series data. [file 1471-2105-6-311-S1.pdf]

We consider an experiment with two technical replicates (external replicate) for each of five concentrations. The chips have a layout with eight blocks and all the probes have an internal replicate with the two probes in a pair positioned below one another. Let x_{rgcj} be the measured $\log_2$ expression value for external replicate r, gene g, channel $c = 1, 2$ and internal replicate $j = 1, 2$. Furthermore, let s_{rgcj} be the standard deviations on the original scale given by the software for each spot. The corresponding standard deviation on a $\log_2$ scale is $w_{rgcj} = s_{rgcj}/[\ln(2)\exp(x_{rgcj}\ln(2))$.

We write the $\log_2$ expression value as a sum of terms (corresponding to multiplicative effects on the original scale). There is a gene level μ_{gc} and a general level ξ_{rcj} dependent on the replicate and the channel. Due to the influence of the spot morphology there is for each gene a term u_{rgj} dependent on the replicate, but not on the channel. Due to the specific binding properties of the two colours there is a term v_{gc} dependent on the channel, but not dependent on the replicate. Finally, there is a measurement error term ϵ_{rgcj}. We denote the variance of the spot term u_{rgj} by σ_s^2, the variance of the channel term v_{rgc} by σ_c^2, and the variance of the measurement error ϵ_{rgcj} by $\sigma^2\omega_{rgcj}^2$, where ω_{rgcj} is a known scaling. Mathematically, the above specification can be written as

$$x_{gcj} = \mu_{gc} + \xi_{rcj} + u_{rgj} + v_{gc} + \epsilon_{rgcj}.$$

We have found that this model is suitable for describing the data looking at one chip only. Below, when comparing two chips, we also consider the extention where the design of the chip is taken into account. Specifically, the spots are divided into 8 blocks and we consider the possibility of having the general level ξ_{rcj} depending on the block also. To standardize the variances of the differences d_g below, we used the scaling $\omega_{rgcj} = w_{rgcj}$.

Before finding the estimates of the variance parameters let us look at the variance of a $\log_2$ foldchange implied by the model. For this we imagine that the general levels ξ_{rcj} have been estimated and subtracted from the data. Then the $\log_2$ foldchange is

$$\begin{aligned} F_{rgj} &= (x_{rg1j} - \xi_{r1j}) - (x_{rg2j} - \xi_{r2j}) \\ &= \{(\mu_{g1} - \mu_{g2}) + (v_{g1} - v_{g2})\} + \epsilon_{rg1j} - \epsilon_{rg2j}. \end{aligned} \tag{1}$$

The non-systematic part consists of the two measurement erors only, and the relevant variance is

$$\sigma^2(\omega_{rg1j}^2 + \omega_{rg2j}^2). \tag{2}$$

By using the external replicate we consider below whether this is the correct variance or whether some extra variance term is needed. We can compare this with the variance when using one colour only and hybridization to two chips to find a foldchange. In the latter case the $\log_2$ foldchange contains a spot term u from each of the two chips as well as a measurement error term from each of the two chips. The variance is therefore

$$2\sigma_s + \sigma^2(\omega_{1gcj}^2 + \omega_{2gcj}^2). \quad (3)$$

We consider below whether this is appropiate using our technical replications, where the true $\log_2$ foldchange is zero, and measured values are

$$F_{gcj}^{one} = (x_{1gcj} - \xi_{1cj}) - (x_{2gcj} - \xi_{2cj}) = u_{1gj} - u_{2gj} + \epsilon_{1gcj} - \epsilon_{2gcj}. \quad (4)$$

We now consider the estimation of the variances σ^2, σ_s^2, and σ_c^2 based on a single chip. We leave out the replication number r in the formulae below. The estimation is based on the following differences

$$\begin{aligned} d_g &= (x_{g11} - \xi_{11}) - (x_{g12} - \xi_{12}) - (x_{g21}) - \xi_{21}) + (x_{g22} - \xi_{22}) \\ &= \epsilon_{g11} - \epsilon_{g12} - \epsilon_{g21} + \epsilon_{g22}, \\ d_g^u &= (x_{g11} - \xi_{11}) - (x_{g12} - \xi_{12}) + (x_{g21}) - \xi_{21}) - (x_{g22} - \xi_{22}) \\ &= 2(u_{g1} - u_{g2}) + \epsilon_{g11} - \epsilon_{g12} + \epsilon_{g21} - \epsilon_{g22}, \\ d_g^v &= (x_{g11} - \xi_{11}) + (x_{g12}) - \xi_{12}) - (x_{g21} - \xi_{21}) - (x_{g22} - \xi_{22}) \\ &= 2(v_{g1} - v_{g2}) + \epsilon_{g11} + \epsilon_{g12} - \epsilon_{g21} - \epsilon_{g22}, \end{aligned}$$

with variances

$$V(d_g) = \sigma^2 s_g^2, \quad V(d_g^u) = 8\sigma_s^2 + \sigma^2 s_g^2, \quad V(d_g^v) = 8\sigma_c^2 + \sigma^2 s_g^2,$$

where $s_g^2 = (\omega_{g11}^2 + \omega_{g12}^2 + \omega_{g21}^2 + \omega_{g22}^2)$. To obtain estimates we replace the general levels ξ_{cj} by median values over the appropiate set of probes, estimate σ^2 by the average of d_g^2/s_g^2, estimate σ_s^2 by the average of $[(d_g^u)^2 - \sigma^2 s_g^2]/8$, and estimate σ_c^2 by the average of $[(d_g^v)^2 - \sigma^2 s_g^2]/8$. The estimates can be seen in Table 1.

We next turn to an evaluation of the reproducibility in the external technical replicate. Our model predicts that the foldchange (1) has the variance (2) for technical replicates. To test this we consider the difference of the $\log_2$ foldchange calculated on two different chips, that is,

$$d_g^{fc} = [(x_{1g1j} - \xi_{21j}) - (x_{1g2j} - \xi_{22j})] - [(x_{2g1j} - \xi_{21j}) - (x_{2g2j} - \xi_{22j})].$$

conc.	σ	$\sigma\bar{w}$	σ_s	σ_c	median fc
1/3	0.10	0.10	0.12	0.36	0.53
	0.13	0.12	0.12	0.38	0.52
1	0.09	0.09	0.13	0.18	1.54
	0.09	0.08	0.11	0.18	1.45
2	0.09	0.08	0.14	0.17	2.53
	0.09	0.08	0.14	0.18	2.61
3	0.09	0.08	0.12	0.17	4.03
	0.09	0.08	0.12	0.18	4.10
4	0.10	0.08	0.13	0.19	4.60
	0.10	0.08	0.12	0.19	4.32

Table 1: Standard deviations for the various random terms in the $\log_2$ foldchange. Included in the last column is the median foldchange.

If (2) is correct the mean of

$$(d_g^{fc})^2 - \sigma_1^2(\omega_{1g1j}^2 + \omega_{1g2j}^2 - \sigma_2^2(\omega_{2g1j}^2 + \omega_{2g2j}^2) \qquad (5)$$

should be zero. A positive value indicate that there is some extra variance not accounted for by the model. The numbers can be seen in Table 2, where the measurement variance $2\sigma^2\bar{w}^2$ (see (2)) has been included for comparison. The values in Table 2 support the model prediction that the variance for technical replicates is given by (2).

conc.	extra variance	$2\sigma^2\bar{w}^2$
1/3	-0.001	0.024
1	-0.001	0.015
2	0.003	0.012
3	-0.004	0.012
4	-0.003	0.012

Table 2: Extra variance for technical replicates as given by the mean of (5). The last column is included for comparison.

We can also check if the model prediction (3) for the variance when using a one colour system is correct. For this we use the differences F_{gcj}^{one} in (4). The mean of

$$(F_{gcj}^{one})^2 - \sigma_1^2\omega_{1gcj}^2 - \sigma_2^2\omega_{2gcj}^2 \qquad (6)$$

should be comparable to $2\sigma_s^2$. The values are given in Table 3. Using a global median centering as above we find that the mean of (6) is larger than $2\sigma_s^2$. However, when a centering is made for each of the 8 blocks on the chip the two variances become comparable in size.

conc.	one colour variance	one colour variance, block	$2\sigma_s^2$
1/3	0.093	0.056	0.027
1	0.057	0.053	0.029
2	0.101	0.044	0.040
3	0.048	0.030	0.027
4	0.046	0.033	0.031

Table 3: Variance in a one colour system. In the first column a global median centering is used, and in the second a median centering for each of the 8 blocks is used. the last column gives $2\sigma_s^2$ for comparison.

Batch replication: Tabel 4 viser at der foregaar noget som vi ikke forstaar. Den ekstra varians ved run 1 er ok, men for stor ved run 2, og i begge runs er one colour variansen alt alt for hoej i forhold til $2\sigma_s^2$. Enten boer man nok finde en forklaring eller lave et nyt batch experiment.

run	extra	$2\sigma^2\bar{w}^2$	one	one, block	$2\sigma_s^2$
1	0.001	0.014	0.552	0.471	0.044
2	0.026	0.011	0.561	0.499	0.056

Table 4: Batch replication. Note that *one colour variance* is much bigger than $2\sigma_s^2$, which is different from the within batch replication.
